# The tragic paradoxical effect of telemedicine on healthcare disparities- a time for redemption: a narrative review

**DOI:** 10.1186/s12911-023-02194-4

**Published:** 2023-05-16

**Authors:** Motti Haimi

**Affiliations:** 1 Clalit Health Services, Tel Aviv, Israel; 2 Rappaport Faculty of Medicine, Technion, Haifa, Israel; 3 School of Public Health, Faculty of Social Welfare & Health Sciences, University of Haifa, Haifa, Israel; 4 Health Disparities Working Group, International Society for Telemedicine and E-Health (ISfTeH), Basel, Switzerland

**Keywords:** Telemedicine, Telehealth, Health Disparities, Accessibility, Paradoxical Effect

## Abstract

**Background:**

Telemedicine has become more convenient and advantageous due to the rapid development of the internet and telecommunications. A growing number of patients are turning to telemedicine for health consultations and health-related information.

Telemedicine can increase access to medical care by removing geographical and other barriers. In most nations, the COVID-19 pandemic imposed social isolation. This has accelerated the transition to telemedicine, which has become the most commonly utilized method of outpatient care in many places.

Telehealth can assist resolve gaps in access to healthcare services and health outcomes, in addition to its primary function of boosting accessibility to remote health services.

However, as the benefits of telemedicine become more apparent, so do the limitations of serving vulnerable groups. Some populations may lack digital literacy or internet access. Homeless persons, the elderly, and people with inadequate language skills are also affected. In such circumstances, telemedicine has the potential to exacerbate health inequities.

**Aim and methods:**

In this narrative review (using the PubMed and Google scholar database), the different benefits and drawbacks of telemedicine are discussed, both globally and in Israel, with particular focus paid to special populations and to the telehealth usage during the Covid-19 period.

**Findings:**

The contradiction and paradox of using telemedicine to address health inequities yet sometimes making them worse is highlighted. The effectiveness of telemedicine in bridging access to healthcare inequities is explored along with a number of potential solutions.

**Conclusions:**

Policy makers should identify barriers among special populations to using telemedicine. They should initiate interventions to overcome these barriers, while adapting them to the needs of these groups.

## Introduction

Telehealth uses communication technologies to exchange information and deliver healthcare services by healthcare professionals, where the participants are separated by geographical distance [1]. By providing health services at a distance, telemedicine enables providers and patients to overcome geographic and other barriers to medical care [2].

Telemedicine has developed in recent years into a practical and secure method for people to get medical advice and information about their health. The quick advancement of internet and communications technology has sped up this trend [3].

During the 2019 COVID-19 pandemic, social distancing became mandatory. This encouraged clinical practices across the world to transition rapidly to telemedicine, and it quickly became the predominant mode of outpatient care delivery.

Telemedicine is now essential and is here to stay. It enables medical practitioners to increase the scope of their direct and urgent medical support services. It enables access to healthcare anywhere there is a communications network [4, 5]. Given that it meets patient requirements and preferences in a way that in-person clinical appointments cannot, it is probable that telemedicine will continue to play a significant role in the delivery of healthcare [6].

There are many benefits in using telehealth, especially in non-emergency everyday care and in cases where services do not involve direct patient-healthcare supplier contact [7]. In addition to its main benefit in promoting ease of access to distant health services, telehealth can also help address disparities in access to healthcare facilities and improve health outcomes [8, 9].

The needs of vulnerable groups, however, become more difficult to meet as the usefulness of telemedicine grows. Although telehealth services have the potential to promote more equitable access to the health care system, particularly for those who live in remote areas, it is unknown how much these services are actually used by different populations, and its implications for promoting the principle of equity are unclear.

Some populations have unique challenges accessing telemedicine. These include individuals with limited digital literacy, those who lack access to digital devices or reliable internet service, individuals experiencing homelessness, and those with limited language proficiency [10].

Furthermore, impediments to receiving telehealth services are more likely to affect members of racial and ethnic minorities, people with lower incomes and education levels, and people who live in rural areas.

Today, internet access is widespread in most countries around the world. However, gaps in access may still be observed across a variety of variables, such as age, gender, race, ethnicity, income brackets, and education levels. While some disparities have declined over the past two decades, many of them are increasing, especially for low-income populations [11–13].

Additionally, some telediagnosis systems simply focus on access problems while ignoring how people in vulnerable communities interpret and understand the information provided. This could exacerbate the health inequities that already exist [14].

## Methods

### Search terms

The following keywords were used to search PubMed and Google Scholar for this narrative review: telemedicine, telehealth, telecare, e-health, elderly, old, COVID-19, SARS-CoV-2, health inequalities, health disparities, advantages, disadvantages, barriers, e-health literacy, digital divide, Israel.

### Selection criteria

Only English-language publications that were published in scholarly journals or organizations between 2020 and 2022 were included.

All types of articles were considered, including original articles, reports of randomized clinical trials, observational studies, and editorials or essays by key opinion leaders.

As with any narrative evaluations, a selection bias cannot be entirely disregarded.

## Findings

### Advantages of telemedicine

Telemedicine offers the opportunity to provide clinical services at a distance, thereby solving geographic and additional obstacles to medical care [2]. It enables providers to offer immediate medical support, making healthcare more accessible and efficient in any location that has a telecommunications infrastructure.

By reducing the need to travel for medical care, telemedicine improves healthcare efficiency and accessibility. It makes it possible to use a range of communication techniques, including video, writing communication, and online translation. Additionally, it has been demonstrated to enhance patient outcomes.

Better long-term care management has also been demonstrated to be made possible by the use of telemedicine. Additionally, it provides a fresh and new way to communicate with medical professionals and find out about health issues.

In a number of ways, telehealth improves patient satisfaction. Better access to care is its primary benefit. By eliminating the need to travel and miss work, it also lessens stress. These elements also translate to greater patient convenience [15].

Telehealth technologies are increasingly being adopted and applied as an efficient and cost-effective means for providing and gaining access to quality health care services. These services are becoming an appealing tool to use worldwide [16].

By using telehealth, clinicians can extend their reach, connecting to remote patients, and usually are able to manage more patients than traditional care models would typically allow. With the increased access achieved by telemedicine, both physicians and patients can collaborate in attaining their therapeutic objectives, especially in home and hospice care settings. The use of telemedicine also has the capability to assist patients become more engaged in their healthcare strategy, increase their independence and compliance [17–19].

By improving access to healthcare in underserved areas, telemedicine improves access to care for all patients, regardless of their location. Telehealth has made it possible to offer services in specialized domains, in particular. A wide range of specialties, including dermatology, cardiology, pediatrics, psychiatry, neonatology, and neurology (stroke), now provide telemedicine possibilities [20].

The use of telemedicine technology also allows for remote monitoring of health and physical status. It can be used by elderly patients to alert their caretakers of changes in activity, falls, or for patients with chronic conditions. With this kind of care, elderly people can stay in their homes for extended periods of time. Based on the preferences of the patient, doctors can design the optimal treatment. Reduced patient expenses are additional advantage [21].

In one study, patients with chronic conditions were shown to be fascinated and curious about utilizing telemedicine, regardless of their age or health status, and described high contentment and satisfaction [22].

Telehealth has been previously reported [23] to be comparable to face-to-face care, in most cases. It may even have better outcomes in cases such as mental assessment and treatment, rehabilitation consultation, anti-coagulation management, and nutrition consultation in older adults.

From the health care organization perspective, telemedicine can reduce health care expenditures by decreasing medication misuse, unwarranted emergency department visits, and prolonged and recurrent hospitalizations. It can improve the flow of data from health records and provide better coordination of care among health care providers at disparate locations [16].

Furthermore, telemedicine has the potential to lower health inequities by providing people in rural locations or places with a shortage of medical professionals' access to care.

### Disadvantages and limitations of telemedicine

Despite the promise of telemedicine to enhance accessibility to healthcare, its adoption has been inconsistent, for several reasons. These include inadequate reimbursement, licensing obstacles, absence of sufficient infrastructure, and opposition to implementing change [24, 25].

There are also drawbacks to telehealth. One is that the medical professional must make a diagnosis without being able to conduct a thorough physical examination and, in certain cases, without even seeing the patient. This can jeopardize patient safety. Physicians in telemedicine setting face various difficulties and challenges [26], but they have developed several methods that help them make accurate diagnoses, including the use of non-medical factors [27]. These means enable health providers to retain an acceptable level of patient safety, even in pediatric tele-triage settings [28].

Technical obstacles [26, 29], security breaches, and regulatory impediments were also reported. Additional critics of telehealth argue that it may adversely affect continuity of care, and that the online interactions are impersonal and even alienating [29, 30]

By removing barriers to care, telehealth has a great potential to reduce health inequities. Unfortunately, lack of access to broadband internet and lack of digital literacy limit this potential. The usage of telehealth is significantly hampered for those who encounter technological challenges, limited e-health literacy (defined as the capacity to use and comprehend online health services), operational challenges, and technical issues [31, 32].

Broadband internet availability varies significantly by demographic class, particularly in rural areas and among different racial and ethnic groupings. The principal platform for home e-health services, the internet, is less accessible to socially weaker populations. Lack of internet connectivity may worsen health inequities because the internet serves as the fundamental platform for telehealth applications.

The possibility of widening inequities among vulnerable people increases as the healthcare system becomes more virtual. These groups have less access to the knowledge and tools required for effective telemedicine use, and their baseline health outcomes are lower. Affected populations include members of ethnic or racial minorities, immigrants, patients who live in rural regions, people with disabilities, elderly people, and patients with limited language competence or have poor levels of digital literacy or income [10, 33, 34]. The COVID-19 pandemic has made these communities' access to care even more difficult [34].

### Telehealth during the Covid-19 era

The COVID-19 pandemic resulted in many challenges for the health care system. Patients and providers had to quickly adapt to telehealth models to prevent and reduce the transmission of COVID-19. This resulted in a rapid transfer to telehealth solutions in both inpatient and outpatient settings [35].

Telemedicine has emerged as the "hottest" and most well-liked technology area worldwide thanks to the Covid-19 pandemic [36]. The rate of growth in telehealth utilization accelerated with the start of the COVID-19 pandemic. The New York Times' headline from April 20, 2020, "10 Years of Change in One Week': Telemedicine on Fast Track" is a stark example of the coronavirus pandemic's impact on the global telemedicine industry.

Telemedicine provided a crucial extra benefit during the pandemic by making it possible to receive medical care without running the danger of contracting an infection through contact with others and without violating government-mandated home quarantine restrictions.

Unfortunately, the use of telemedicine during the pandemic was not distributed equally across the population [37]. The COVID-19 pandemic has increased health disparities for vulnerable, weak, and marginalized populations across the world. The pandemic produced a disproportionate threat for ethnic minority groups to contract COVID-19, because of their higher representation in positions that do not allow working from home. Furthermore, racial and ethnic minority populations have higher rates of comorbid diseases like obesity, diabetes and hypertension. This puts them at higher risk of mortality when infected with the virus.

Elderly people and those with pre-existing medical illnesses are more likely to acquire a severe form of COVID-19 disease [38, 39], and these people are also among the demographics that are least likely to have access to telemedicine.

According to Qian et al. [40], patients at the greatest risk of COVID-19 infection used telemedicine the least due to racial/ethnic, language, and low economic level differences.

Despite the health gaps that became apparent during the COVID-19 pandemic, this period revealed that many services can be operated from a distance (such as education, shopping, etc.), including telemedicine, if so desired.

### Telemedicine and the elderly population

Telehealth has the potential to enhance equality in care for the elderly population as well, especially in the COVID-19 era, but sadly it can also further exacerbate disparities [41–44].

Many elderly persons with complicated medical conditions had limited access to care even before the COVID-19 outbreak. The disruption of acute-care services in medical institutions, including early hospital discharges, postponement and rescheduling of non-urgent elective procedures and outpatient appointments, and staff reorganization, has had an impact on older people with chronic health conditions in addition to the scaling back of community services [45, 46].

Due to fewer clinic visits, transportation restrictions, and other societal changes brought on by the pandemic, the COVID-19 pandemic made the problem of access to care even more difficult. Additionally, the COVID 19 pandemic's lack of social connection and physical activity may have contributed to a decline in the mental and physical health of vulnerable elderly people [47].

Several studies show that older people find using telemedicine complex. The obstacle is not necessarily the ease of access, but age-related obstacles such as lack of skill and experience in using telemedicine, lack of health literacy, lack of support from others, and physical and cognitive disabilities [45]. Older adults also tend to face barriers in trust and confidence when using telehealth [48–53].

Nevertheless, the myth that older people lack technology devices and internet connections has been disproven. In fact, most of them do have such accessibility, although they find it difficult to use. According to some reports, most older adults (70%) have and utilize a computer, smartphone, or tablet with internet access at home, but, when it comes to the use of telehealth, there is restricted reach among this population [54].

But when given the chance, many elderly people are able to use telemedicine successfully, particularly when convenience is the main priority and when specialized equipment is set up so they may engage in telehealth from home [55–60].

In a recent systematic review [61], we found that while older patients may benefit most from using telehealth visits, which increase their access to care, ironically not enough telehealth solutions are tailored for this particular demographic or focused on their requirements. This was particularly true during the COVID-19 pandemic, when the necessity of such remedies was unequivocally shown.

### Telemedicine and health disparities in Israel

Ethnic, linguistic, and social minorities in Israel's diverse society encounter numerous obstacles to using telehealth effectively. There are signs of widespread and widening disparities in health care and the health state of Israel's population, despite major efforts made by the Ministry of Health of Israel and increased government spending in the health sector. The substantial variance in health conditions and disease prevalence among various sub-groups in Israel is also a result of the country's pronounced income and financial disparities.

Overall life expectancy in Israel is high by OECD standards. Still, several population groups have inferior health levels, such as non-Jews; people living outside the major population centers, at the geographical periphery; and individuals with low socio-economic status. Inequalities affect e-health literacy and internet access, thus encouraging a digital divide, which influences the accessibility to telemedicine and exacerbates health disparities [62].

The healthcare system has recently undergone changes that have increased the privatization of healthcare services and increased spending on private healthcare. In light of this trend, it is now more crucial than ever to examine how Israel's telehealth services relate to the concepts of equality and equity.

A 2017 Israeli study [63] that looked at telemedicine use among adult members of a major Israeli health maintenance organization (HMO) found a negative correlation between telemedicine use and age. Only 43% of people 75 and older reported using these services, compared to 60–63% of people in the 55–74 age range and 69% of people in the 45–54 age range.

According to Jaffe et al. [64], certain hurdles to using technology may eventually go away since digitalization is becoming a part of everyday life and because older individuals' access to and usage of digital technology has expanded considerably in the past ten years.

Another Israeli study [65] looked into "e-health literacy"—the relationship between age, literacy levels, and accessing health information online. Their research revealed that younger, more educated persons had better levels of e-health literacy than older, less educated people. Additionally, individuals in this category were more passionate internet users overall. In comparison to the respondents who were less familiar with e-health, they employed more search techniques and conducted more thorough information analysis. Additionally, individuals in the highly e-health literate group found more beneficial information during their information search, including cognitive, instrumental (self-managing health needs, improved health behaviors, and better utilization of health insurance), and interpersonal (interaction and relationship with the doctor) advantages.

"The association of e-health literacy with background attributes indicates that the Internet reinforces existing social differences," the authors concluded. New disparities in the field of digital health information are brought about by the Internet's rising use and sophistication, as well as the ensuing gains among highly literate users.

The Israeli government unveiled a brand-new national initiative in March 2018 under the name "Digital Health as an Engine of Growth." Through this program, six government ministries are collaborating to develop Israel's digital healthcare sector into a national growth engine and a center for cutting-edge medical technology on a global scale. Both academics and medical professionals are encouraged by the initiative to conduct extensive research in the area of digital health. The Israeli government established this partnership in recognition of the importance of telemedicine to the efficiency of its healthcare system. Israel is intended to become a global leader in telemedicine and digital health solutions. As a leader and an example for health systems, Israel also seeks to offer future ideas that can be adopted globally.

The enormous potential of telemedicine has long been recognized in Israel, which has given it national priority status, massive funding, suitable legislation and regulations, and a supportive environment for partnerships between health organizations, research institutes, start-up enterprises, and independent researchers. The Israeli Ministry of Health recently stated that its goal is "to generate a leap in the health system that will enable it to become sustainable, advanced, innovative, renewed, and constantly improving, by optimally leveraging the information, and communication technologies available to the entire Israeli population" [66].

In addition, during the last two years, the Israeli Ministry of Health founded a special "telehealth community" of professionals involved in the telemedicine field. It also established several working groups in different specialties (such as tele-pediatrics and tele-geriatrics), to define working practices and make recommendations for the Ministry in the field of telemedicine.

The COVID-19 pandemic has boosted and accelerated telemedicine use and implementation in Israel, as it has in other developed nations. The pandemic has created an opportunity to advance telemedicine more vigorously and create a variety of services more quickly than anticipated. This quickening of telemedicine fits with the aim of promoting and supporting digital services, making them sustainable, advanced, inventive, and constantly developing and improving, as was previously mentioned.

The thoughts and attitudes of adults on the employment of telemedicine during the COVID-19 lockdown were the subject of a second Israeli study [67]. The findings indicated that most users of telemedicine during that time preferred it and were happy with its performance. In addition, nearly 80% of the participants reported willingness to use telemedicine in the future.

Another recent study evaluated Israeli pediatricians' use of telemedicine before and throughout the pandemic's initial lockdown period [68]. The authors claim that during the initial COVID-19 lockdown, primary care pediatricians significantly boosted their usage of telemedicine technologies. However, they anticipated that use would decline once the outbreak was over.

The utilization of remote health services and obstacles to use among Israel's minority Arab population were discussed in a recent Brookdale Institute paper [69]. Most respondents had used at least one of the researched services during the previous year and had no fundamental objections to using telehealth services. Phone calls were determined to be the most accessible and simple to use of the services examined by the study. By incorporating more phone conversations into healthcare services, the entire community would have remote access without facing socioeconomic or cultural restrictions.

In terms of other services, the Brookdale study found that individuals with the most need and potential benefit—including parents of young children, older people, and the chronically ill—were the groups that used them the least. Additionally, it was discovered that the likelihood of using telehealth services (other than phone calls) increased with increasing levels of education and literacy. The authors of the study also noted that encouraging patients to use telehealth services and providing guidance and technical support by family members and healthcare professionals may raise familiarity and awareness of these services among the Arab community and increase their use.

The ultra-Orthodox Jewish community, which has distinct cultural, religious, and demographic traits, is another distinctive population in Israel. This society upholds a high standard of deference to religious leaders. They have little access to secular stimuli and incentives from outside their insular societies. The community and its values are extremely important in these communities. The ultra-Orthodox keep separate, independent educational systems that are centered on conventional religious subjects. They stay away from—and often even forbid—using televisions, computers, and the internet to limit exposure to secular media [70, 71].

Understandably, the use of telehealth solutions in the ultra-Orthodox populations poses many challenges, due to their limited access to virtual communication as well as their unwillingness and hesitation to participate in this new mode of treatment. To these communities, virtual treatment is strange and unfamiliar, and may be viewed as conflicting with their religious principles. For these reasons, online virtual consultations may act as a barrier to treatment in some cases. This may cause distinct issues for non-religious professionals trying to treat patients from this population. These concerns existed prior to the COVID-19 pandemic, but have worsened during the COVID-19 period, when face-to face medical encounters were significantly restricted [72].

Despite the challenges and barriers stated above, a recent paper [73] describes virtually home-based therapy for ultra-Orthodox young women who had previously been hospitalized for eating disorders, during the COVID-19 pandemic in Israel. The study indicated that this mode of treatment was appropriate, satisfactory and agreeable to certain families.

## Discussion

"Health disparities" is a term used to describe inequality in health indicators, such as prevalence, frequency, morbidity, and mortality, which is brought on by unequal access to resources like money, freedom, knowledge, or social capital or by unequal distribution of health services [74].

Often, disparities in health arise due to the unequal distribution of these goods in general society. That is, they are the result and reflection of existing social gaps. For example, obesity caused by an unhealthy diet is common among the lower classes in Western countries, and reflects their difficulty in purchasing healthy food, a phenomenon that has been named the "paradox of food insecurity as a cause of obesity," or the "obesity and poverty paradox" [75, 76].

However, occasionally advancements in the field of medicine, like the creation of new, effective interventions, can itself worsen or even create new inequities in health. The "profit" from the intervention is allocated differently in society, which is a phenomenon known as "intervention-generated inequality" [77].

There are several benefits to telemedicine, especially when services don't require face-to-face interactions between patients and doctors. It also makes a significant contribution to the development of healthcare in neglected areas. This technology attempts to improve everyone's access to care regardless of where they are while minimizing in-person visits and the usage of healthcare resources. As a result, the medical system might even save costs [8, 15].

Another significant advantage is providing broad access to caregivers, particularly in situations of chronic illnesses [78]. Telehealth incorporates a wide variety of practices and specialties and encompasses interactions between patients and providers through numerous modes such as telephone, e-mail, video chats or conferences, the internet, and remote devices [29].

The COVID-19 epidemic presented the healthcare sector with a number of difficulties and challenges. Health care providers quickly shifted to telehealth systems in many regions to maintain the safe and efficient delivery of care for patients with and without COVID-19. The pandemic loosened the legislative and financial restrictions that had previously prevented the broad use of telehealth, hastening the rate of development in this area.

Patients and providers were compelled to quickly adapt to telehealth models in order to prevent and minimize the spread of COVID-19 [35]. The general public developed a fundamental need and demand for telehealth applications and solutions at this time, making it possible for people to consult their doctors for guidance, direction, support, and advice on their medical issues. Telemedicine proved essential for treating COVID-19 sufferers as well as healthy people who had to stay in quarantine [36].

Telemedicine will undoubtedly play a significant role in the delivery of healthcare going forward, even after the pandemic, as it can address service shortages and cater to patients' desires and requirements in a way that in-person clinical appointments cannot [6].

However, if this technology is applied and accepted inequitably, the transition to telemedicine would exacerbate the significant underlying gaps in health-care that the pandemic has revealed. Access to telemedicine poses special difficulties for those with low digital literacy, restricted access to digital devices or high-speed internet, homelessness, aging populations, and limited English (or other language) competence [10, 14].

A study in the field of tele-psychiatry [Connolly et al.,^79^] found that although most patients and healthcare providers welcome telehealth, disparities in health outcomes and healthcare delivery persist across racial and ethnic minorities, individuals with lower incomes and socioeconomic status, older adults, and residents of rural areas [80].

The COVID-19 pandemic has given us the chance to appreciate the enormous significance of digital health initiatives for the general community while also realizing that special populations had restricted access to standard medical care. During the pandemic, telehealth usage significantly expanded and developed at a faster rate, which has attracted a lot of interest. However, concerns have also been expressed regarding the impact of increased use on socioeconomic and demographic health inequities [81].

By connecting isolated and rural people with medical professionals, telehealth has the potential to significantly reduce the access gap to healthcare. Sadly, it appears that people who might most likely benefit from telehealth also have the biggest barriers to accessing it. As the healthcare system becomes more virtual, gaps among disadvantaged groups may worsen since they already have poorer health outcomes and less access to the resources needed for effective telemedicine utilization [61].

Offering health equity in telehealth entails providing everyone with the chance to receive the medical treatment they require and deserve, regardless of their socioeconomic situation, and making the necessary modifications to digital literacy, technology, and equipment. This will make it easier for telehealth providers to reach the underserved groups who most need their services [82–85].

While there are many obstacles to health equity, physical access to care is likely the biggest one that might be quickly removed with increased telehealth use. However, attempts to expand access to care through telehealth are severely constrained by limitations in broadband internet access and digital literacy [86].

It is not enough to access telehealth even though ordinary internet access has extended across racial and ethnic groups without noticeable discrepancies (although not completely distributed by age, income, and population density). For users to access video conferences from their homes, a large bandwidth is required. For successful telehealth appointments, patients also need to be digitally (or e-health) literate. They must comprehend the operation of telehealth platforms and the specialized terminology used during remote medical consultations. Particularly for older persons, those with less education, and members of racial or ethnic minorities, this digital literacy is challenging [87].

In this essay, different facets of telemedicine were examined, including its potential to lessen disparities and, on the other hand, its drawbacks and difficulties that could result in a rise in health disparities.

This narrative study aims to highlight the odd paradox surrounding the impact of telemedicine and health technologies on health disparities. Digital health and telemedicine technologies aim to improve health, on the one hand. Theoretically, by increasing accessibility and efficiency, they can lessen health inequities. They reduce the need for travel and improve patient outcomes by providing medical support through communication technologies.

On the other side, because those who have the necessary resources can benefit from these technologies more quickly, easily, and effectively than the disadvantaged, telehealth can also exacerbate health disparities.

Health disparities are common in many countries, particularly in access to telehealth. Israel is a unique mélange of minorities, religions, cultures, and populations, some of which have special difficulties in accessing telemedicine. Nevertheless, while disparities might expand on some levels, the knowledge developed since the pandemic has showed us that telehealth technology has the potential to mitigate disparities in a profound way. It does so by improving access to care, and presumably, health outcomes, for a wide range of disadvantaged populations.

In order to promote and encourage ideas that will aid in removing these barriers to healthcare, policymakers worldwide should be aware of the issue raised in this review and take note of the circumstances in other nations.

Telemedicine provided an instant solution to the threats and challenges posed by the COVID-19 pandemic. These findings prompted healthcare officials and practitioners to acknowledge the potential of telemedicine as a reliable, secure, and practical treatment option.

Due to its role during the recent pandemic, the telemedicine revolution is achieving remarkable momentum. Most probably, the practices implemented during this period will be maintained in the future, especially for medical training, routine practice, service delivery, and policies.

To retain the benefits of telemedicine, governments and private organizations must establish additional initiatives and conduct evaluations to address the challenges and the barriers that different sub-groups may experience in accessing these services. Policymakers must identify the obstacles that prevent certain special populations from using telemedicine, and they must then launch suitable interventions to remove these obstacles in a way that is suited to the requirements of these groups.

A trustworthy guarantee of coverage for patients and medical professionals is necessary for telehealth to effectively bridge gaps in access to care. Along with providing enough infrastructure, it's also important to educate and train both patients and doctors. Finally, there has to be a more equitable distribution of technology exposure and digital literacy among various socioeconomic and demographic groups.

In regard to the senior population, as was previously noted [61], suitable and effective digital solutions should be designed expressly for the elderly sub-group, concentrating on their regular everyday requirements and activities rather than just during pandemic situations. For instance, doctors or HMOs can schedule phone calls for routine health checks for older patients.

It is also advised that older patients use simple and basic technological devices (such tablets), which will be provided to them, in order to readily connect with their doctors or other healthcare professionals. Healthcare providers can address this digital divide by giving talks and lectures and showing off telemedicine options. Another choice is to educate and prepare specialized healthcare or technology experts who may go to senior patients and assist them in learning how to use the digital devices, linking them to their distant healthcare providers.

With these interventions, the point of care will move from the doctor's office to the patient's location, fundamentally altering the way that these particular populations access and use health services. The telemedicine paradigm won't be reversed once patients, providers, and policymakers realize and comprehend that it actually works. Instead, it will be incorporated into the medical service system, medical education, and the future of the medical profession.

## Conclusions

Telehealth can assist alleviate gaps in access to healthcare services, as well as enhance health outcomes, in addition to its fundamental benefit of expanding accessibility to remote health services. However, socially poorer groups have less access to the internet and digital devices, which are the main platforms for home e-health services, and lower levels of literacy. Therefore, paradoxically, e-health services can ultimately cause a widening of the health disparities between the various demographic subgroups.

These limitations and disparities in telemedicine usage among various populations should be known to policymakers. To fully realize the enormous promise embodied in telemedicine, they should advocate for specialized actions to bridge these gaps and hurdles. Regulation may be necessary to ensure that everyone has access to internet resources, especially for particular population groups like the elderly and illiterate. Even affirmative action in favor of disadvantaged people or members of minorities in disadvantaged areas might be taken into consideration by governments.

## Data Availability

All relevant data id cited in the references.
